# The Development of an Abattoir-Based Surveillance System in Lao PDR for the Detection of Zoonoses in Large Ruminants: Q Fever and Brucellosis Seroepidemiology as a Pilot Study

**DOI:** 10.3390/ani11030742

**Published:** 2021-03-08

**Authors:** Jarunee Siengsanan-Lamont, Bounlom Douangngeun, Watthana Theppangna, Syseng Khounsy, Phouvong Phommachanh, Paul W. Selleck, Nina Matsumoto, Laurence J. Gleeson, Stuart D. Blacksell

**Affiliations:** 1 Mahidol-Oxford Tropical Medicine Research Unit, Faculty of Tropical Medicine, Mahidol University, Bangkok 10400, Thailand; jarunee@tropmedres.ac (J.S.-L.); Paul.Selleck@csiro.au (P.W.S.); laurence.j.gleeson@gmail.com (L.J.G.); 2 National Animal Health Laboratory, Department of Livestock and Fisheries, Ministry of Agriculture, Vientiane 0102, Laos; bounlom.douangngeun@gmail.com (B.D.); wtheppangna@hotmail.com (W.T.); s.khounsy@gmail.com (S.K.); phou.vong@hotmail.com (P.P.); 3 Sydney School of Veterinary Science, Faculty of Science, University of Sydney, Camden 2570, Australia; nmat3443@uni.sydney.edu.au; 4 Centre for Tropical Medicine & Global Health, Nuffield Department of Medicine, University of Oxford, Oxford OX1 4BH, UK; 5 Lao-Oxford-Mahosot Hospital-Wellcome Trust Research Unit (LOMWRU), Mahosot Hospital, Vientiane 0100, Laos

**Keywords:** seroprevalence, zoonosis, Lao PDR, large ruminants, Brucella, Q fever

## Abstract

**Simple Summary:**

An abattoir based surveillance system was implemented in the Lao People’s Democratic Republic to determine the prevalence of two diseases that can spread between animals and humans: Brucellosis and Q fever. A total of 683 cattle and buffalo samples were collected from abattoirs in six selected provinces between March–December 2019. Laboratory diagnostic tests to detect antibodies against both diseases were performed and the number of animals that tested positive for either disease was relatively low. However, extensive animal movement within the country was also identified, which has the potential to increase the risk of spreading disease within and between countries. Monitoring of high impact animal/human diseases assists pathogen surveillance and the country’s food security. This study highlights the importance of ongoing animal health surveillance and the need to find cost-effective approaches for its long-term sustainability.

**Abstract:**

Although animal health surveillance programmes are useful for gaining information to help improve global health and food security, these programmes can be challenging to establish in developing economies with a low-resource base. This study focused on establishing a national surveillance system initiated by the Lao PDR government using a passive surveillance system of abattoir samples as a pilot model, and to gain information on contagious zoonoses, particularly Q fever and brucellosis, in the large ruminant population. A total of 683 cattle and buffalo samples were collected from six selected provinces of Lao PDR between March–December 2019. Out of 271 samples tested, six samples (2.2%, 95% confidence interval (CI) of 1.0, 4.8) were positive in the Q fever antibody ELISA test. Only one sample (out of 683; 0.2%, 95% CI 0.0, 0.8) tested positive to the Brucella antibody ELISA test. Seroprevalence of these important zoonoses in Lao PDR were relatively low in cattle and buffaloes; however, extensive animal movement within the country was identified which could increase risks of spreading transboundary diseases. The study highlights the importance of ongoing animal health surveillance and the need to find cost-effective approaches for its long-term sustainability.

## 1. Introduction

The animal health services of the Lao People’s Democratic Republic (Lao PDR) are predominantly delivered by the Department of Livestock and Fisheries (DLF) of the Ministry of Agriculture and Forests (MAFF). In this role, DLF is responsible for meeting the national requirements for animal disease surveillance, for example as a member country of the World Organisation for Animal Health (OIE), and a participating country in the OIE South-East Asia and China Foot-and-Mouth (SEACFMD) control program. With increased animal trade within ASEAN countries and with China, there is a need for baseline information on animal health status and more routine data on disease prevalence. Linked to this trade there is also a need to understand more about the impacts of endemic and epidemic diseases on animal production, and potential or actual impacts of zoonotic livestock diseases on public health. However, the implementation of animal disease surveillance activities, both active and passive, is considerably constrained by the resources available to the DLF. Historically disease prevalence information has been an output of project activities supported by international agencies [1].

To address some of the gaps in the animal health information system, the DLF initiated a pilot approach that used passive serological surveillance based on routine abattoir collections across a range of sites. While recognising the inherent constraints on such surveillance information, there is a possibility that it may be fit-for-purpose in the national context in Lao PDR, this at this time being to get a plausible indication of the likely prevalence and potential impact of endemic diseases. One focus of the surveillance pilot study was an assessment of the utility of the approach to estimate the prevalence of two zoonotic diseases, namely Q Fever and brucellosis, in large ruminants. There have been earlier reports on the prevalence of these two diseases [2,3] and so there was some historical information to compare with the outputs of this activity.

## 2. Materials and Methods

### 2.1. Sample Collections

**Figure 1:**
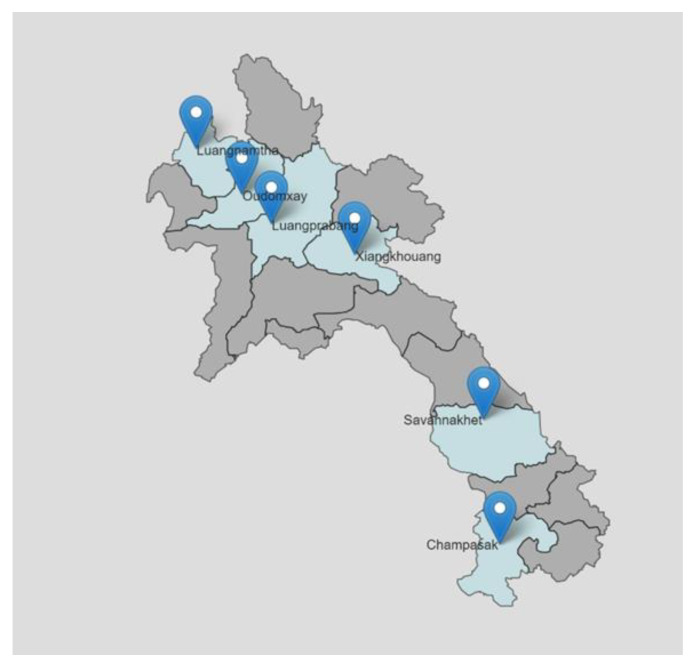
Six provinces included in the abattoir surveillance program.

The surveillance program was conducted in six provinces representing central, northern and southern regions of Lao PDR, namely Luangnamtha (LNT), Luangprabang (LPB), Oudomxay (ODX) (all northern), Savannakhet (SVK) and Champasak (CPS) (southern), and Xiengkhouang (XK; central) (Figure 1). Serum samples were collected from abattoirs by Provincial-level and District-level Agricultural and Forestry Office (PAFO and DAFO) staff. Animal data including place of origin, age, body score (1–5), vaccination and health statuses were recorded in the sample collection form. A sample size of 11 animals per abattoir was determined using the animal sample calculator [4] with the estimated disease prevalence of 20%, diagnostic test sensitivity (Dse) of 99%, confidence level (CL) of 95% and an estimated abattoir herd size (N) of 30. Previous sero-surveillance studies in Lao PDR reported that the prevalence of brucellosis and Q fever were 0.3% and 1.2% in livestock [2]. The expected prevalence of 20% used in the sample size calculation was justified for an endemic disease screening but not limited to brucellosis and Q fever. The abattoir herd size of 30 was based on an observation of animal numbers at an abattoir in Vientiane province which included animals to be slaughtered on the day and those kept in the lairage.

During the three-month trial period, for simplicity up to 10 cattle or buffalo samples, depending on animal availability, were collected twice a month from a single abattoir in each province between March and July 2019 inclusive. An evaluation of the network was conducted in late July 2019 to identify constraints and adjust approaches. From October–December 2019 the sample size of cattle and buffalo was increased to a maximum of 20 samples per collection round (~every 15 days). Ten ml of blood were collected from the jugular vein of abattoir animals by PAFO and DAFO officers and stored in a cooler for transportation back to the offices. Blood samples were centrifuged at 1000–2000× *g* for 10 min to separate serum samples. The serum samples were transferred into labelled cryotubes, stored at 4 °C and submitted by the provinces to the National Animal Health Laboratory (NAHL) in Vientiane by air freight within five days of the collection date. The samples were then registered into the NAHL’s sample database system and stored at −20 °C until being tested. The animal ethics approval of this study was granted by DLF, MAFF.

### 2.2. Sample Testing

The samples were tested for antibodies against *Brucella spp.* using the ID Screen^®^ Brucellosis Serum Indirect Multispecies ELISA (ID.VET, Grabels, France, Cat# BRUS-MS-10P) and antibodies against Q fever using the ID Screen^®^ Q Fever Indirect Multispecies ELISA (ID.VET, France, Cat# FQS-MS-5P). Both ELISAs were performed according to the manufacturer’s instructions provided in the kits. The Sample to Positive Percentage (S/P%) was then calculated using IDSoft ^TM^ software version 5.05 provided with the ID Screen^®^ ELISA kits [5]. The diagnostic cut-off for the brucellosis ELISA was based on the manufacturer’s recommendations where samples were considered negative when S/P % ≤ 110%, doubtful when 110% < S/P% >120% and positive when S/P% ≥ 120%. For the Q Fever ELISA, samples were classified negative when S/P% ≤ 40%, doubtful when 40% < S/P% ≥ 50% and positive when S/P% > 50%. The Dse and diagnostic test specificity (Dsp) of the ID Screen^®^ Brucellosis ELISA were 100% and 99.7% while Dse and Dsp of the ID Screen^®^ Q Fever ELISA were 100% and 100%, respectively [6]. Samples tested positive to the brucellosis ELISA were then confirmed by the Rose Bengal technique (RBT) according to the manufacturer’s instructions as described by OIE [7]. Of the total 683 samples collected, a subset of 271 samples were tested for Q fever antibodies due to the shortage of available diagnostic tests.

### 2.3. Analysis

Descriptive statistical and spatial analyses were performed in Microsoft Excel [8] and R Studio Version 1.2.1335 [9]. Frequency and probability distributions were used to describe the dataset. Apparent and true seroprevalences were estimated using the Wilson method as applied to imperfect tests [10]. Visualisation of animal movement data was generated using the leaflet R package [11]. Fisher’s Exact tests were fitted to compare differences of each factor (origin province, destination province, age, sex, type and collection month) between positive and negative animals using RStudio [9].

## 3. Results

### 3.1. Samples and Logistics

**Figure 2:**
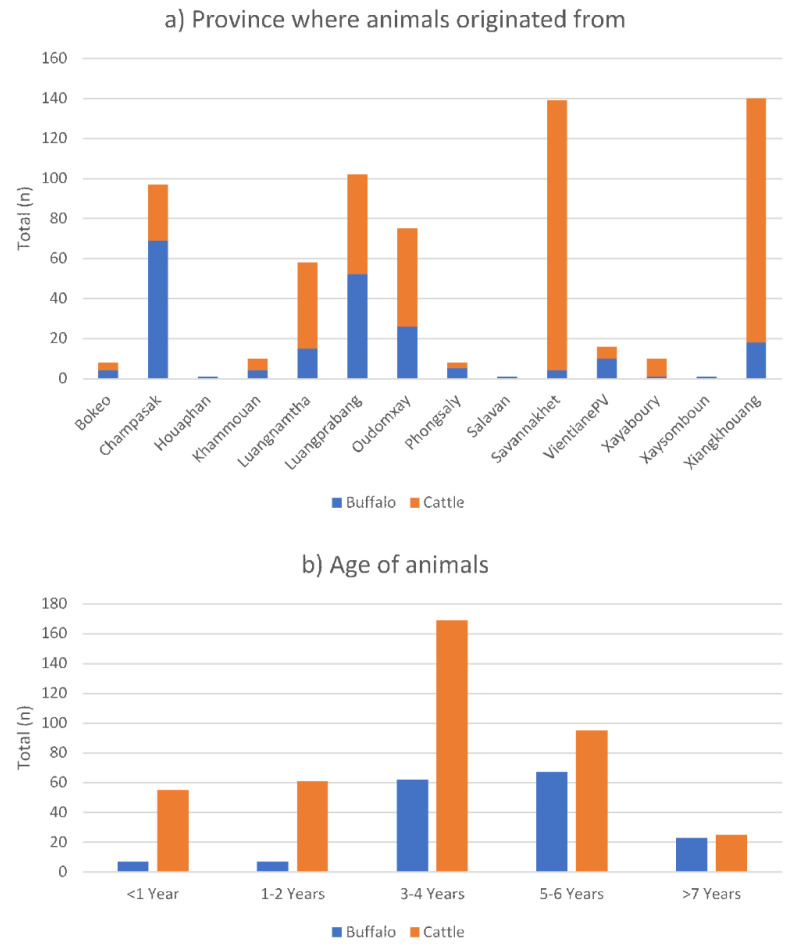
Origin (**a**) and age (**b**) of the animals.

A total of 683 samples were collected during the survey, with approximately 24.9% (*n* = 170) collected from XK and 20.1% (*n* = 143) from SVK province (Table 1 and Table 2). Out of 666 cattle and buffaloes (note: Due to some data on animal origin not being recorded, numbers of total (*n*) were varied.), 21.0% (*n* = 140) and 20.9% (*n* = 139) originated from XK and SVK provinces, respectively. More than 32% (*n* = 69) out of 211 buffaloes were from CPS province (Figure 2a). Only one buffalo and no cattle (*n* = 666) originated from each of Houaphanh, Salavan and Xaysomboune. Most animals in this study ranged in age from 3–4 years (40.5%, 231 out of 571) and 5–6 years (28.4%, *n* = 162) (Figure 2b). More than 98% (*n* = 673) out of 683 animals were recorded as native breeds. DAFO and PAFO staff were able to collect and deliver samples on schedule. Field and laboratory consumables were sourced from Thailand as there was no established supply chain in Laos PDR. Consumables imported to Lao PDR were distributed from NAHL via public transportation to provinces in batches. Transnational supply constraints led to some disruptions of the survey program and resulted in no samples being collected for August and September 2019. The survey resumed in October 2019 and was completed in December 2019. A fixed cost for sending samples from a province to NAHL in Vientiane Prefecture was 310,000 Laos Kip/LAK (~35 USD) per shipment excluding labour costs. On average, field consumables cost 1 USD per sample, while the cost of laboratory tests (ELISA) and consumables was approximately 4 USD per sample per test excluding labour and utility costs.

### 3.2. Q Fever and Brucellosis Serology

A total of six samples (2.2%; 6/271) were Q fever antibody positive and one sample was interpreted as doubtful (Table 2). Samples tested for Q fever antibodies were those collected between March and October 2019. All six positive samples were collected from an abattoir in XK province (five local animals and one originated from LPB). Given the Dse and Dsp of the test were 100%, both apparent and the true prevalence of Q fever were 2.2%, 95% confidence interval (CI) of 1.0, 4.8. Only one sample collected from the LNT province (0.1% out of 683 tested samples) was positive for Brucella antibodies (Table 2). This sample also tested positive in the RBT. The apparent seroprevalence of brucellosis was 0.2%, 95% CI of 0.0, 0.8. Fisher’s Exact tests showed no significant association of the factor (origin province, destination province, age, sex, type and collection month) between positive and negative animals.

### 3.3. Animal Origins and Movements

**Figure 3:**
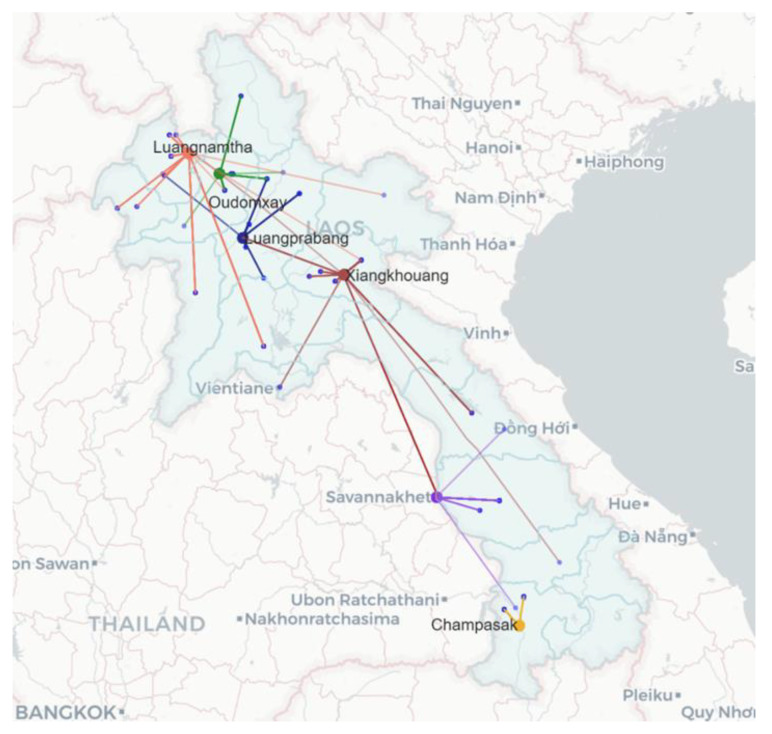
Animal movement map from a source of origin (blue dot) to an abattoir in the six provinces (LNT = orange, ODX = green, LPB = Navy, XK = brown, SVK = violet and CPS = yellow).

Records of animal origins were used to plot an animal movement map (Figure 3) and to qualitatively compare the animal supply data. Abattoirs in XK and LNT provinces received animals from more provinces and longer distances when compared to the other four provinces. One of the longest estimated distances was a buffalo slaughtered in XK province, transported from Salavan province (estimated ~780 kilometres, 14.5 h travel by road [12]). The majority of animals at the abattoirs were local livestock, with the balance being sourced from a range of other provinces (Figure 3). For example local animals accounted for 84.8% (139 out of 164) of samples collected in XK, followed by 5.5% (*n* = 9) from Khammouan province, 3.7% (*n* = 6) from LPB and SVK provinces and 1.2% (*n* = 2) from Vientiane Prefecture. Out of 93 samples collected in LNT, 62.4% (*n* = 58) were local animals following by 15.1% (*n* = 14) and 10.8% (*n* = 10) from Vientiane Prefecture and Xayaboury, respectively. Abattoirs in CPS (*n* = 96) and LPB (*n* = 85) provinces sourced their animals locally within their provinces.

## 4. Discussion

This study presents evidence for low antibody prevalence for both brucellosis and Q fever in cattle and buffalo slaughtered in select northern, southern and central provinces of Lao PDR. Due to the presence of a “doubtful” range of the IDScreen^®^ diagnostic kits, it was highly unlikely that these tests have a Dse or Dsp of 100%. This study also presents information regarding the long-distance transport of animals to slaughter in Laos, which raises wider questions regarding inadvertent dispersal of major livestock diseases, including Foot and Mouth Disease (FMD). The numbers of animals slaughtered at an abattoir each day varied depending on market demand. Nampanya et al. [13] reported that the estimated numbers of cattle and buffalo slaughtered per year in both XK and LPB were 5000–7000 head. Based on these figures, the average numbers of animals slaughtered per day were 14 (5000/365) to 20 (7000/365), assuming activity every day. Taking this information into account, for a province like LNT which had the smallest human population among the selected provinces [14] smaller numbers of livestock slaughtered per night were expected. Almost half of the samples in this study were collected in only two provinces (XK and SVK) and therefore the results should be interpreted with caution due to this potential bias. PAFO and DAFO reported low numbers of animals slaughtered at abattoirs per day in the other four provinces. For a future surveillance program, sample sizes should be justified to better represent populations in each province.

The logistics system in Lao PDR was sufficient to support the programme, and public transport services between provinces (bus and plane) were utilised as the primary logistics of the survey network. Regardless of sample numbers, one-way shipping from a province to NAHL was capped (at ~35 USD per shipment). If the number of samples per shipment was low, the cost-effectiveness of the logistics was questionable. When adding costs of sample collection and transportation, labour (field and laboratory staff per diem/salary), consumables and other utilities at the national level, long-term sustainability could be difficult to achieve. Further application and analysis of an expanded programme might indicate how a limited but targeted seroepidemiology programme could be conducted to monitor the prevalence of important animal diseases in the large ruminant population. There are long-term challenges to implementing animal disease surveillance programs, especially if laboratory support is required to confirm diagnosis. However, there might be value in the future in examining the use of the fast growing hi-speed mobile networks and existing staff social network chat groups to share disease information and to implement a combination of syndromic surveillance, early reporting and targeted sampling.

The numbers of cattle or buffalo sampled at abattoirs in this study could represent proportions of livestock populations in these provinces. XK and SVK provinces historically have had the highest density of cattle compared to other provinces [15]. SVK province also had the largest buffalo population reported with up to 19% of the Laos buffaloes followed by CPS province (13%) [16]. In our study, XK province had a higher proportion of cattle samples compared to buffalo samples, while CPS province collected a large proportion of buffalo samples. Data also revealed that some animals travelled long distances from their origin provinces to their destination abattoir. Livestock movement is generally driven by high demand in the biggest city provinces and also neighbouring countries [17]. Previous studies on animal movement reported that livestock travelled from Thailand via Lao PDR destined to Vietnam and China, mainly through XK and LNT provinces respectively [18,19]. In this study, there was no record of animals originating from Thailand. However, both XK and LNT provinces received livestock with more variety and from more distant provinces than others despite a small number of livestock slaughtered per night in LNT province as mentioned above. The livestock movement routes from other provinces to LNT and XK provinces in this study were fairly similar to the movement maps reported by Smith et al. [18]. This may represent a high demand for livestock for an export trade rather than for local consumption in these two provinces. Without effective disease control measures, animal movement for slaughter could increase the risk of spreading transboundary animal diseases e.g., FMD [13].

Although Douangngeun et al. [2] reported that Q fever was not widely distributed in Lao PDR as their study detected Q fever positive in only some Northern provinces, a later survey reported seropositive goats in SVK (Southern) province [3]. In 2006, Vongxay et al. [20] reported that the seroprevalences of Q fever and Brucella antibodies in cattle and buffalo were 4% and 0.2%, respectively. A goat survey in 2016–2017 in Lao PDR by Burns et al. [3] revealed a similar Q fever seroprevalence of 4.1% but a higher *Brucellosis* spp. seroprevalence of 1.4%. In neighbouring Thailand, a survey of Q fever antibodies in dairy cattle in a Northern province revealed a seroprevalence of 5% [21]. Earlier studies of *Brucellosis* spp. in Thailand reported no seropositive to *B. abortus* antibodies in a dairy cattle survey in 2007 [22] and 1% seropositive against *B. melitensis* antibodies in goats between 2008–2010 [23]. Similar to previous studies, seroprevalences of Q fever and brucellosis in this study were relatively low. All Q fever seropositive samples in this study were cattle from XK province except for one animal that originated in LPB. However, the distribution of Q fever in Lao PDR cannot be concluded at this stage. The low seroprevalence of brucellosis may be contributed by the high manufacturers recommended S/P% cut point (≥120%) of the ELISA test [2] or local livestock raising practices of a non-intensive, pasture grazing system [24]. The impacts of brucellosis [25] and Q fever [26] on human health in Southeast Asia were not well-established with some publications on case reports in neighbouring countries (e.g., Thailand and Vietnam). Only one study in Lao PDR on seroprevalence of *B. melitensis* and *C. burnetii* of 30 hospital patients with endocarditis was identified [27]. Given brucellosis and Q fever are present in Lao PDR, there is a potential risk to human health especially for those who have close contact with infected animals [26]. Current epidemiological studies and publications of Q fever and brucellosis statuses in livestock in Lao PDR and other Southeast Asian were limited and often subjected to specific population and demographics which may not represent the true disease status. It highlighted the need to continue and broaden monitoring and surveillance activities of zoonoses and other high impact diseases. To gain an in-depth understanding of zoonosis and animal disease epidemiology, an extensive countrywide longitudinal study is required. In depth disease investigations (including active surveillance) targeting high risk areas and/or populations should also be considered. However, these are expensive undertakings and also difficult to implement with the human and physical resources available.

In low resource countries, it is recognised that launching an animal disease surveillance program at the national level can be challenging [28]. An abattoir surveillance program provides an option for monitoring and detecting disease presence in a population [29]. The animal supply data would suggest that the sero-surveillance as conducted can provide useful information on the likely prevalence level of Q fever and brucellosis in the province where the samples are collected. However, the prevalence identified by such a program may not represent the true prevalence of the population as selections of animals to abattoir are often subjective [29]. Tracing back positive animals would help identify high risk areas for further investigation and control measures. Cumulative abattoir survey data over a period of time would also provide useful disease information about the geographic distribution of disease [29]. Major constraints identified in this study included a lack of financial and human resources and limited infrastructure. Current animal disease surveillance programs were solely funded by international agencies or foreign aid projects with objectives that may not fully align with the country’s priorities. Limited government budget resulted not only in limited numbers of personnel across the animal health sector at both regional and national levels (e.g., DAFO, PAFO, laboratory staff, etc.) but also in inadequate consumables, equipment and infrastructure maintenance. Field and laboratory staff were often well trained but stretched across multiple international funded projects. Recommendations included encouraging ownership among the host government sectors, engaging stakeholders to reduce redundancy and exploring alternative approaches to reduce costs and increase sustainability.

## 5. Conclusions

Developing and implementing a routine animal disease surveillance system in a low resource setting proved challenging. An abattoir surveillance program could provide an alternative system to collect animal health data. The design of such a surveillance program not only needs to balance expected scientific outputs and field practicality given various limitations but also to take into account cost effectiveness and sustainability. This study highlighted the importance of ongoing animal health surveillance, discussed lessons learnt, and provided recommendations for future animal health surveillance activities.

## Figures and Tables

**Table 1:** Q Fever and brucellosis Ab ELISA results.

No	Date	Type	Age	Place of Origin	Q Fever %S/P	Q Fever
1	29 March 2019	Cow	1–2 Year	Navarn, Phaxay, XK	96%	Positive
2	24 March 2019	Cow	3–4 Year	Soy, Paek, XK	58%	Positive
3	6 May 2019	Cow	3–4 Year	Done, Kham, XK	145%	Positive
4	5 June 2019	Cow	5–6 Year	Lath-ngon, Paek, XK	55%	Positive
5	24 October 2019	Cow	4–6 Month	SVK	41%	Doubtful
6	24 October 2019	Cow	4–6 Month	Phoukhoune, LPB	61%	Positive
7	24 October 2019	Buffalo	4–6 Month	Lardyaiy, Phoukood, XK	70%	Positive
**No**	**Date**	**Type**	**Age**	**Place of Origin**	**Brucellosis %S/P**	**Brucellosis**
1	9 December 2019	Cow	4 Year	Luk52 Phonhong, Vientiane Prefecture.	155%	Positive

**Table 2:** Summary of the serum samples and test results in six provinces.

Destination Province	Total Samples Collected		Q Fever Ab ELISA		Brucellosis Ab ELISA	
	Cattle	Buffalo	Total Tested	Positive	Total Tested	Positive
CPS	28	68	19	0	96	0
LNT	63	33	28	0	96	1 ^¶^ (1.0%)
LPB	36	49	35	0	85	0
ODX	60	33	26	0	93	0
SVK	140	3	57	0	143	0
XK	142	28	106	6 (5.7%)	170	0
Total	469	214	271	6 (2.2%)	683	1 (0.1%)

^¶^ Positive in Rose Bengal Test.

## Data Availability

No new data was created or analyzed in this study. Data sharing is not applicable to this article.
